# Metformin Reduces Vascular Assembly in High Glucose-Treated Human Microvascular Endothelial Cells in An AMPK-Independent Manner

**DOI:** 10.22074/cellj.2021.7212

**Published:** 2021-05-26

**Authors:** Carolina Silva, Ilda Rodrigues, Sara Andrade, Raquel Costa, Raquel Soares

**Affiliations:** 1. Department of Biomedicine, Unit of Biochemistry, Faculty of Medicine, University of Porto, 4200-319 Porto, Portugal; 2. i3S, Institute of Research and Innovation in Health, University of Porto, Porto, Portugal; 3. IPATIMUP, Institute of Pathology and Molecular Immunology, University of Porto, Porto, Portugal

**Keywords:** AICAR, AMPK Signaling, Compound C, Endothelial Cells, Metformin

## Abstract

**Objective:**

The aim is to examine the effect of metformin in human microvascular endothelial cells exposed to high glucose (HG) concentration and compare them with the effects of other 5' adenosine monophosphate-activated protein kinase (AMPK) modulators under the same condition.

**Materials and Methods:**

In this experimental study, human microvascular endothelial cells (HMECs) were treated with 15 mM metformin, 1 mM 5-aminoimidazol-4-carboxamideribonucleotide (AICAR) and 10 mM compound C in the presence of 20 mM glucose (hyperglycemic condition). Migration, invasion and proliferation were evaluated as well as the capillary-like structures formation. Moreover, the expression of angiogenic genes was assessed.

**Results:**

Metformin significantly inhibited vessel formation and migration, although it did not change HMECs proliferation and invasion. In addition, metformin significantly reduced collagen formation as evidenced by histological staining. Concomitantly, expression of several genes implicated in angiogenesis and fibrosis, namely *TGFß2, VEGFR2, ALK1, JAG1, TIMP2, SMAD5, SMAD6* and *SMAD7*, was slightly upregulated. Immunostaining for proteins involved in ALK5 receptor signaling, the alternative TGFß signaling pathway, revealed significant differences in SMAD2/3 expression.

**Conclusion:**

Our data showed that metformin prevents vessel assembly in HMECs, probably through an AMPK- independent mechanism. Understanding the molecular mechanisms by which this pharmacological agent affects endothelial dysfunction is of paramount importance and paves the way to its particular use in preventing development of diabetic retinopathy and nephropathy, two processes where angiogenesis is exacerbated.

## Introduction

In recent years, the incidence of type 1 diabetes mellitus (Fig T1DM) has increased worldwide, contributing to a significant increase in overall rates of diabetes morbidity and mortality (1). Vascular complications present in the vast majority of patients with T1DM, are responsible for a considerable part of morbidity rate (2). It is known that inflammatory changes in the blood vessel wall lead to a dysfunction in endothelial and smooth muscle cells resulting in vascular disease (3). Endothelial cells are particularly vulnerable to hyperglycemia (4). Thus, uncontrolled hyperglycemic state, common in diabetic patients, leads to increased release of factors that favor endothelial dysfunction (5). In turn, endothelial dysfunction is a potential contributor to the pathogenesis of vascular disease in DM (6), resulting in reduced bioavailability of nitric oxide (7). Studies performed in humans, animals and cells showed that endothelial dysfunction is maintained even after normal glycemia is achieved, a concept designated by metabolic memory (8). Therefore, as vascular complications are the major cause of morbidity in diabetic patients, understanding the molecular events that occur in endothelial dysfunction is mandatory.

Previous studies of our group revealed that endothelial cells isolated from T1DM mice kidney and heart exhibited a distinct gene expression profile involving AMPK pathway, a major cell energy regulator (9). AMPK pathway can be modulated by pharmacological agents like metformin. In agreement, several reports suggested that stimulating AMPK signaling leads to endothelial dysfunction improvement (10). Moreover, AMPK signaling activation improves insulin sensitivity and reduces the risk of T2DM (11).

Metformin, one of the most commonly used antihyperglycemic drugs against T2DM (12), is a known activator of AMPK and has also been studied as an adjuvant for T1DM treatment. Metformin could activate AMPK indirectly, through inhibition of mitochondrial complex I and increment of the AMP/ ATP ratio, or directly, by α subunit phosphorylation (13). Although the molecular mechanisms of metformin action are not completely elucidated, this compound can be therapeutically successful in other pathological conditions as well. In cancer, for instance, metformin exerts anti-proliferative effects as demonstrated *in vitro* and *in vivo* (11). 

Metformin was shown to target angiogenesis as well, interfering with endothelial function and attenuating the production of proangiogenic and inflammatory factors like metalloproteinases (MMP’s), adhesion molecules, namely intercellular adhesion molecule 1 (ICAM-1) and vascular cell adhesion molecule 1 (VCAM-1), tumor necrosis factor α (TNFα) and nuclear factor-κB (NF-κB) (14-18).

Given the wide use of metformin in diabetic patients, as well as the relevance of AMPK signaling pathway in diabetic complications, the present study aimed to elucidate how AMPK modulators affect HMECs. To address this, HMECs cells were cultured with AMPK agonists, metformin and AICAR, and an AMPK antagonist, compound C, in the presence of 5.5 mM (normoglycemic) or 20 mM (hyperglycemic) glucose, and cell proliferation, migration and assembly into capillary-like structures, as well as expression of angiogenic genes were examined.

## Materials and Methods

### Cell culture and *in vitro* treatments

Human microvascular endothelial cells (HMECs, ATCC, UK) were cultured in RPMI 1640 medium (Sigma-Aldrich, Portugal) supplemented with 10% fetal bovine serum (FBS, Sigma-Aldrich, Portugal), 1.176 g/L sodium bicarbonate (Merck, Germany), 4.76 g/L HEPES, 1% penicillin/streptomycin (Sigma-Aldrich, Portugal), 1 mg/L hydrocortisone >98% (Sigma-Aldrich, Portugal), and 10 μg/ml endothelial growth factor (EGF, Sigma-Aldrich, Portugal). Cells were kept at 37˚C in a humidified 5% CO_2_ atmosphere and the experiments were accomplished between passages 3 and 6. Assays were performed in serum-free media supplemented with glucose at two different concentrations: 5.5 mM [low glucose (LG)] or 20 mM D-Glucose [high glucose (HG)] (Sigma-Aldrich, Portugal). Cells were maintained under these conditions for 24 hours before treatment incubation. Cells were then treated with15 mM metformin (Sigma-Aldrich, Portugal), 1 mM 5-aminoimidazol-4- carboxamideribonucleotide (AICAR, Sigma-Aldrich, Portugal) and 10 µM compound C (Sigma-Aldrich, Portugal).These concentrations were selected based on a preliminary viability assay using different concentrations of metformin (10, 20, 30, 40 and 50 mM), AICAR (0.2, 0.5, 0.75, 1.0, 1.25 and 1.5 mM) and compound C (5, 7.5, 10, 12.5 and 15 µM), done based on previously published reports (16, 19-21). AICAR and metformin were dissolved in ultrapure water, whereas compound C was solubilized in dimethyl sulfoxide (DMSO, Merck, Germany). The working solutions were prepared in PBS and then added to respective treatment media.

### Ethical issues

This study was approved by Department of Biomedicine, Faculty of Medicine, University of Porto, Portugal.

### Cell viability

Cell viability was examined by3-(4,5-dimethylthiazol-2- yl)-5-(3-carboxymethoxyphenyl)-2-(4-sulfophenyl)- 2H-tetrazolium (MTS) assay (Cell Titer 961 Aqueous ONE Solution Reagent, Promega, Madison, EUA). HMECs (1×10^5^ cells/mL) were incubated with glucose at two concentrations, for 24 hours. Next, the cells were incubated with metformin, AICAR and compound C for 24 hours. Cell cultures were then incubated with 20 µL MTS according to the manufacturer instructions. Color development was quantified at 492 nm. The concentration of compounds used in all subsequent experiments was defined based on the MTS results in order to exclude possible cytotoxic effects. These concentrations were identical to the ones described in the literature (16, 19-21). Results are expressed as percentage of the control.

### Bromodeoxyuridine proliferation assay

HMECs (1×10^5^ cells/mL) were cultured in serumfree media supplemented with glucose at two different concentrations, in 96-well microplates for 24 hours. Cells were then incubated with treatments in the presence of bromodeoxyuridine (BrdU) at a final concentration of 0.01 mM for another 24 hours. Cells were then fixed and incubated with anti-BrdU antibody for 90 minutes. Detection was performed using the colorimetric BrdU Proliferation Assay kit (Roche, Germany), according to the manufacturer’s instructions. Optical density was measured at 450 and 650 nm and the results are expressed as percentage of the control.

### Injury assay

Injury assay was performed as described by Liang et al. (22). Cells were plated, maintained at 37˚C in a humidified 5% CO_2_ atmosphere until confluence and then, incubated with the two different concentrations of glucose for 24 hours. Cell cultures were then injured by the pipette tip, which left a void space. The wells were photographed at 200X amplification, and the treatments were added to serum-free media and incubated for 24 hours. The wound closure was determined by subtracting the wounded area measured after 24 hours, from the initial void space (FIJI software, National Institutes of Health, USA).

### Matrigel assay

Matrigel assay was performed in 96-well microplates coated with 50 µl of Matrigel Basement Membrane Matrix (BD Matrigel™, BD-Biosciences, Belgium) per well. HMECs, previously incubated with 5.5 or 20 mM glucose, were harvested in complete medium over the Matrigel layer. Two hours later, the medium was removed and the treatments were added. Cell growth was monitored for 18 hours. Tube formation was observed and quantification was performed by vessel counting in each well using a phase contrast microscope (Nikon, UK), at ×200 magnification.

### Collagen synthesis evaluation in cell culture

Production of collagen by cells was analyzed by Sirius Red histologic assay, as described by Pinheiro et al. (23). Briefly, cells were cultured with low and high concentrations of glucose for 24 hours and then, incubated with compounds (metformin, AICAR and compound C) for an additional 24 hours. Subsequently, HMECs were fixed with 4% p-formaldehyde for 15 minutes at room temperature (RT), washed with distilled water and stained with Sirius Red solution for 1 hour. Wells were washed with acidified water (5%), and incubated with 0.1 N NaOH for 30 minutes, and color development was measured by reading the absorbance at 550 nm using a microplate reader.

### Invasion assay

Invasion assay was accomplished in CorningBioCoat™ Matrigel Invasion Chamber (Transwells, Corning Inc., Corning, USA) according to the manufacturer’s instructions. Basically, following 24 hours under hyperglycemic condition, HMECs (2.5×10^4^ cells/ mL) were harvested on inserts, initially hydrated with complete medium. The lower chambers were filled with RPMI medium containing 10% FBS. After 24 hours incubation with compounds, the non-invasive cells were detached by a cotton swap. Cells enclosed to the lower surface membrane insert were fixed, stained and counted under a microscope from sixteen randomly chosen fields in each well. The mean number of the cells per field was recorded.

### Western blot

Proteins were extracted from homogenates of treated HMECs cultures and quantified by BCA protein assay kit (Thermo Scientific, USA). Then, 15 µg of total protein was separated by electrophoresis and transferred to nitrocellulose membrane (Biorad, USA). The membranes were the incubated with primary antibodies [phospho-SMAD5 (1:500); total SMAD5 (1:500); phospho-SMAD2/3 (1:500); total SMAD2/3 (1:1000) and TGFβR1 (1:500)], and then incubated with secondary horseradish-peroxidase (HRP)-coupled antibodies (1:5000, anti-rabbit, HRP NA934V or 1:5000, anti-mouse, HRP NA931V). Antibodies were dissolved in BSA solution, containing 4% of BSA in 0.1% TBS-T. Detection was performed using enhanced chemiluminescence (ECL) kit (Biorad, USA) and relative intensity of different proteins expression was calculated and normalized against intensity of stainedfree gels (Biorad, USA).

### Quantitative real-time polymerase chain reaction assays

Total RNA was extracted from HMECs after incubation with compounds for 24 hours, using NZYol isolation reagent (NZYtech, Portugal). Briefly, the cells were harvested with 1 mL of the reagent, homogenized and incubated for 5 minutes at RT. For the phase separation, we added 200 µL of chloroform to the tubes, and the tube was incubated for 2-3 minutes at RT and centrifuged. The aqueous phase was transferred to a new tube and RNA precipitation was performed by adding 500 µl cold isopropanol. RNA pellet was washed with 75% ethanol, air dried for 10 minutes and resuspended in RNase-free water. RNA was quantified by NanoDrop.

The cDNA was synthesized by RevertAid H Minus First Strand cDNA Synthesis Kit (Thermo Scientific, USA), and then, 1.5 µL of cDNA sample was used for each polymerase chain reaction (PCR) assay. Gene amplification was performed as previously established (9) under the following conditions: pre-incubation (95˚C for 600 seconds), amplification (95˚C for 10 seconds; specific temperature of each primer; 72˚C for 10 seconds 45 cycles) and melting (95˚C for 10 seconds; (AT+10)˚C for 60 seconds and 97˚C for 1 second); primers used for human *ALK1, JAG1, SMAD5, SMAD6, SMAD7, TGFBR1,TIMP2, TGFβ2, VEGFR2* and *β-ACTIN* are shown in Table 1. Samples were analyzed by Light Cycler 96 thermal cycler (Roche, USA) and quantified by the ∆∆CT method. All genes expression values was normalized against *β-ACTIN* expression values, as a commonly used housekeeping gene.

### Statistical analysis

GraphPad Prism 6.0 Software (GraphPad Software Inc., CA, USA) was used for data analysis and the results are expressed as mean ± SEM, with a confidence interval of 95% and P<0.05 considered significant. Experiments were performed in triplicate and analyzed by one-way ANOVA and Bonferroni post hoc test. Student t test was used for two group analyses with P<0.05 considered significant.

## Results

### Effect of AMPK modulators on HMECs viability

**Fig.1:**
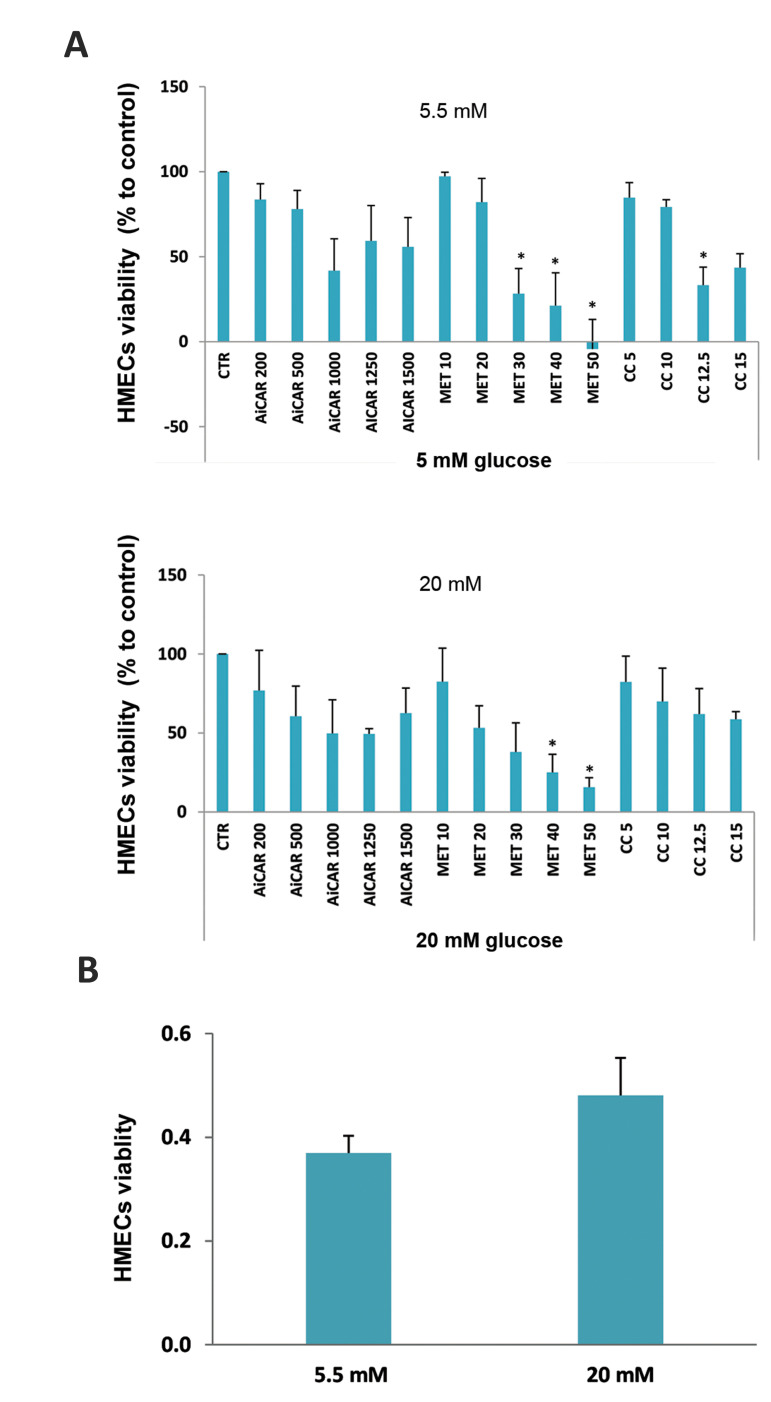
Cell viability evaluation in confluent HMECs cultures using MTS assay. **A.** No significant cytotoxicity was found following treatment with AICAR at a concentration range of 200-1500 µM, metformin (MET) at concentrations of 10-50 mM and compound C (CC) at concentrations of 5, 10 and 15 µM, in most of the doses tested. Results are expressed as percentage of control and are fold-increase relative to normoglycemic (5.5 mM) control cell cultures. Control bars (CTR) refer to cultures under the same conditions of glucose but without incubation with tested compounds. Three independent experiments were performed in triplicate with identical results. *P<0.05 vs. CTR under identical glucose conditions. **B.** Cytotoxicity evaluation in confluent HMECs cultures using MTS assay exposed to 5.5 (CTR5.5) and 20 mM (CTR20) of glucose. No significant difference in cell viability was found between the two concentrations.

To examine the effect of AMPK pathway in endothelial cells, we first analyzed the effect of metformin, AICAR and compound C, three AMPK modulators, at different concentrations in HMECs exposed to low and high concentrations of glucose. The analysis showed a dose-dependent reduction in cell viability for these three agents under both glucose conditions. Incubation with 30 to 50 mM metformin led to a significant decrease in HMECs cell viability at both glucose concentrations, indicating a toxic effect of this agent at these concentrations (Fig .1A). Furthermore, incubation of 5.5 mM glucose-treated HMECs cells with 12.5 µM compound Cresulted in a cytotoxic effect. No significant difference was observed between the two glucose concentrations used for any of the treatments tested (Fig .1B). We, therefore, used the nontoxic concentrations of 1 mM AICAR, 15 mM metformin and 10 µM compound C in the following experiments; the selected concentrations were in agreement with the literature.

**Table 1:** Primer sequences used in HMECs cells exposed to medium containing either 5.5 or 20 mM of glucose, and incubated with AICAR, metformin or compound C


Genes	Primer sequence (5ˊ-3ˊ)

*ALK1*	F: CAACATCCTAGGCTTCATC
	R: TCTCTGCAGAAAGTCGTAG
*β-ACTIN*	F: AGAGCCTCGCCTTTGCCGAT
	R: CCATCACGCCCTGGTGCCT
*JAG1*	F: ACTACTACTATGGCTTTGGC
	R: ATAGCTCTGTTACATTCGGG
*SMAD5*	F: CCAGTCTTACCTCCAGTATTAG
	R: TCCTAAACTGAACCAGAAGG
*SMAD6*	F: CCCATAGAGACACAAAAATCTC
	R: GTAAGACAATGTGGAATCGG
*SMAD7*	F: CAGATTCCCAACTTCTTCTG
	R: CTCTTGTTGTCCGAATTGAG
*TGFBR1 (ALK5)*	F: AGACAATGGTACTTGGACTC
	R: GTACCAACAATCTCCATGTG
*TGFB2*	F: AGATTTGCAGGTATTGATGG
	R:ATTTCTAAAGCAATAGGCCG
*TIMP2*	F: GGCCTGAGAAGCATATAGAG
	R: CTTTCCTGCAATGAGATATTCC
*VEGFR2*	F: GCCATGTGGTCTCTCTGGTT
	R: GCCGTACTGGTAGGAATCCA


### Effect of treatment of HMECs with metformin, AICAR and compound C on HMECs proliferation, migration and invasion 

**Fig.2:**
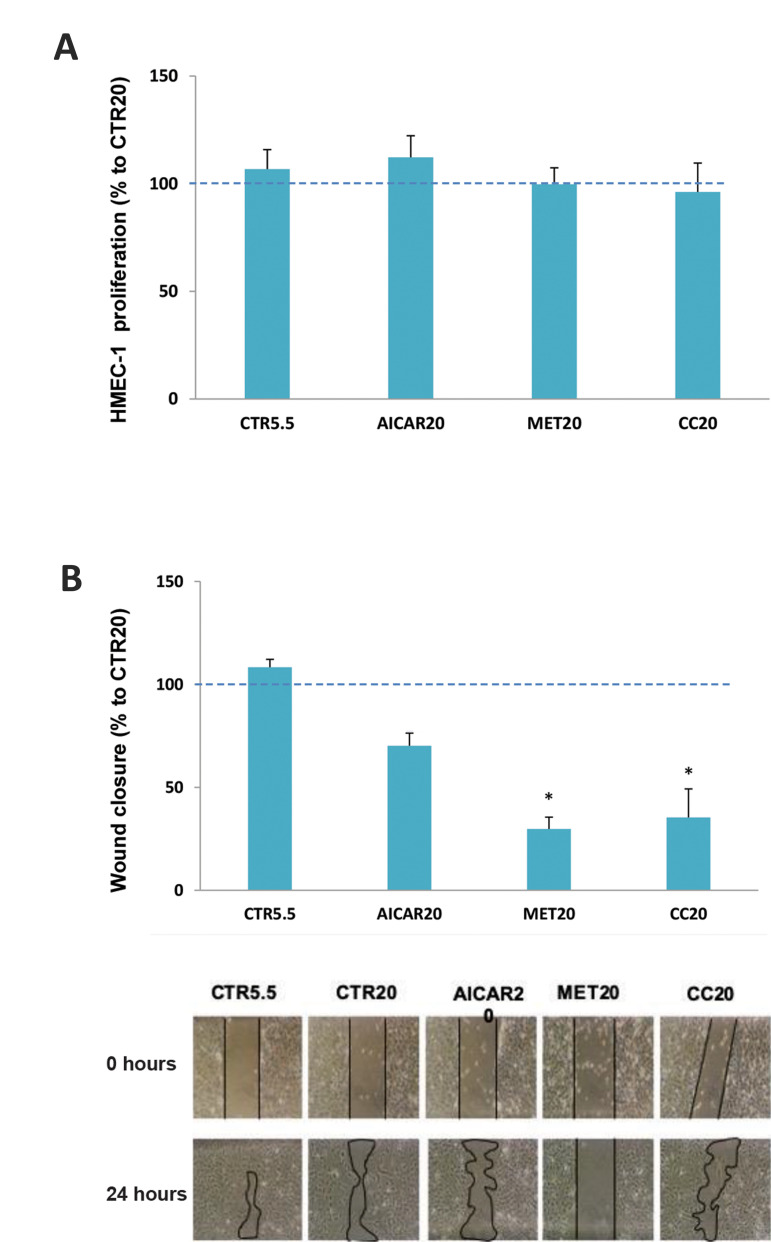
Proliferation and migration of HMECs were evaluated after incubation with AICAR, metformin (MET) or compound C (CC). **A. **Cell proliferation was assessed by BrdU incorporation assay. Cell proliferation was not significantly reduced by incubation with any of the compounds tested. Results are expressed as percentage of high glucose (HG) control. Three independent experiments were performed in triplicate with identical results. **B.** Cell migration was visualized by injury assay after 24 hours of incubation. A significant reduction of cell migration to the damaged areas was found after incubation with CC. Pictures are representative of three independent studies (magnification: ×200). CTR5.5 bar represents 5.5 mM glucose-incubated HMECs in the absence of compounds. *; P< 0.05 vs. CTR20.

In order to determine the effect of these compounds on HMECs proliferation, the BrdU assay was performed. As shown in Figure 2A, no difference in HMECs proliferation was observed between the two glucose concentrations. Furthermore, incubation with 1 mM AICAR, 15 mM metformin or 10 µM compound C in the presence of high concentration of glucose (Fig .2A) did not significantly affect HMECs proliferation.

Moreover, upon incubation with metformin and compound C, a significant decrease in HMECs migration was verified in comparison with the control under the same glucose condition (Fig .2B). A minor reduction in cell invasion was found up on treatment with 1 mM of AICAR, though it did not reach statistical significance.

### Effect of AICAR, metformin and compound C incubation on vessels formation

**Fig.3:**
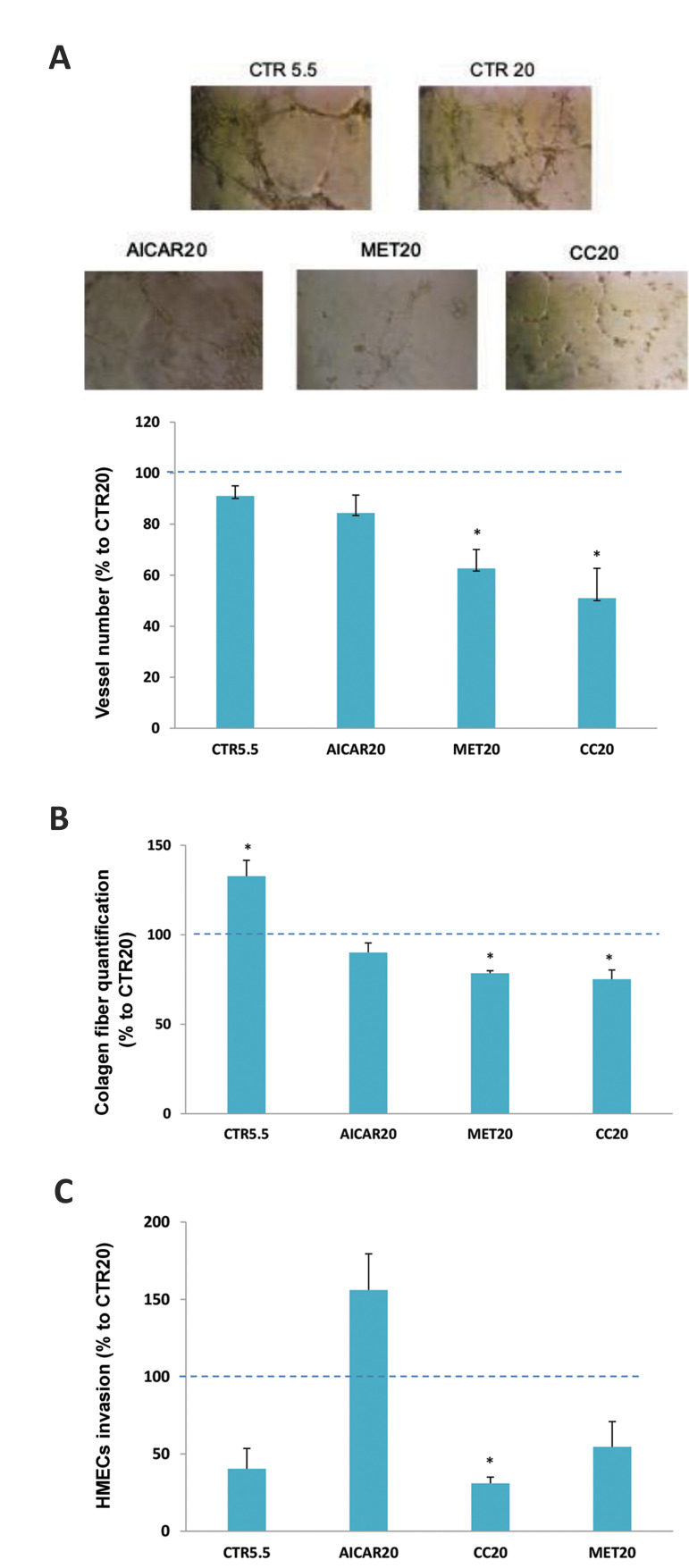
Analysis of vessel formation, cellular invasion and collagen fiber after treatments with compounds. **A.** Assembly of capillary-like structures after incubation with AICAR, metformin (MET) or compound C (CC). Results are expressed as percentage of CTR20 (dotted line); CTR5.5 bar represents 5.5 mM glucose-incubated HMECs left untreated. The number of cord structures was recorded on an inverted microscope. Vascular assembly was reduced in a significant manner after incubation with MET20 as compared to untreated cells (CTR20). *P<0.05 vs. CTR20. Pictures are representative of three independent studies. **B. **Quantification of histological staining for Sirius red in HMECs cells treated with AICAR, MET or CC in the presence of 20 mM glucose. **C. **Analysis of cellular invasion after treatments with AMPK modulators compounds by transwells assay. Significant reduction in HMECs treated with CC20 when compared to control at the same glucose concentration (CTR20, dotted line). Bars represent percentage of control (CTR20, dotted line, magnification: ×200).

To further examine the role of the three AMPK modulators in the development of capillary structures, we established HMECs cell cultures on a 3D basement membrane matrix and monitored the growth of vascular structures. Interestingly, hyperglycemic conditions slightly reduced the formation of capillary-like structures. Incubation of 20 mM glucose-treated cells with each AMPK modulator further reduced the capacity of HMECs to assemble into vessels; this decrease was statistically significant for 15 mM metformin and 10 µM of compound C (Fig .3A). 

The assembly of endothelial cells within blood vessels tructures is strongly dependent on the formation of a basement membrane. Therefore, we next investigated whether the studied pharmacological agents influenced the formation of collagen under hyperglycemic conditions using Sirius Red staining. Treatment with HG resulted in a reduction of collagen formation (Fig .3B). When compared with the control at the same glucose concentration, the incubation with compound C (10 µM) and metformin (15 mM) resulted in a significant decrease of collagen formation by HMECs. However, incubation with 1 mM AICAR did not affect the collagen synthesis by HMECs. Only incubation with 10 μM compound C significantly affected the invasive behavior of HMECs as evidenced by transwells assay (Fig .3C).

### Effect of AICAR, metformin and compound C on angiogenic-related genes

**Fig.4:**
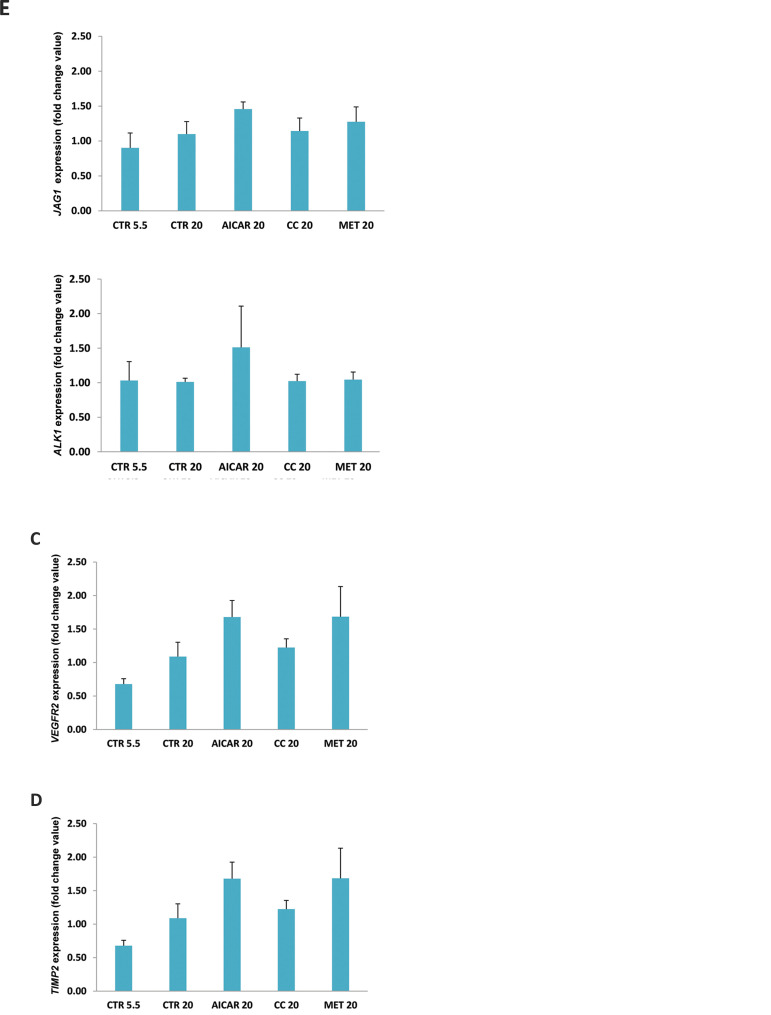
Expression analyses of genes associated with angiogenic process after treatments with compounds. Expression analyses of **A.** *TGFB2*, **B.** *SMAD5*, **C.*** VEGFR2*,** D.** *TIMP2*, **E. ***JAG1 *and **F. ***ALK1* genes in HMECs after incubation with AICAR, metformin (MET) and compound C (CC) in 20 mM glucose for 24 hours. CTR5.5 and CTR20 bars represent gene expression of 5.5 and 20 mM glucose treated HMECs, respectively.

We next performed quantitative real time PCR in order to investigate whether AMPK modulators interfered with angiogenic gene expression in HMECs under HG conditions. Recent experiments of our group showed that *TGFβ2, SMAD5, ALK1, JAG1, VEGFR2 and TIMP2* genes, which are known to play a role in angiogenesis and fibrosis, presented imbalanced expression in endothelial cells from T1DM mice (9). We, therefore, analyzed the expression of these transcripts in HMECs. Incubation with 20mM glucose did not result in significant differences in expression of these genes (Fig .4). 

**Fig.5:**
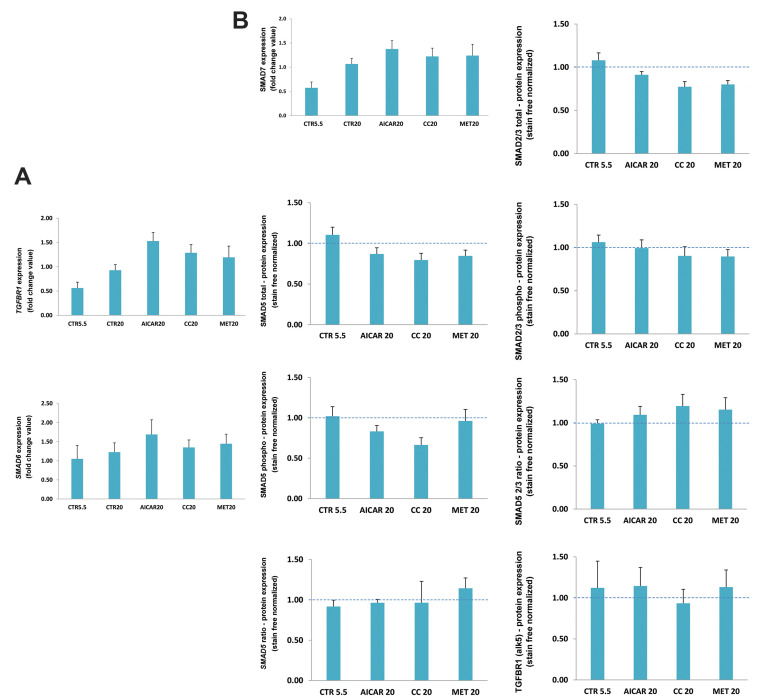
Gene and protein expression analyses in cells after treatment. **A.** Expression analyses of *TGFBR1, SMAD6* and *SMAD7 *genes in HMECs after incubation with AICAR, metformin (MET) and compound C (CC) in 20 mM glucose for 24 hours. CTR5.5 and CTR20 bars represent gene expression of 5.5 and 20 mM glucose treated HMECs, respectively. **B. **Analysis of protein expression by Western Blot after treatments with AMPK modulators for 24 hours. None of the treatments significantly changed the active forms of proteins analyzed.

In general, incubation of cells with AMPK-modulating agents led to an increase in expression of these six genes analyzed in comparison to HG control (Fig .4A-F). Particularly, incubation with metformin resulted in a slight upregulation of *TGFβ2, TIMP2, ALK1, JAG1, SMAD5* and *VEGFR2*, although it did not reach statistical significance. Then, we examined the expression of specific genes of TGFβ signaling pathway like *SMAD6, SMAD7* and *TGFβR1*.Although metformin treatment led to a slight increase in expression of these transcripts, it was not statistically significant (Fig .5A).

To confirm these findings, we analyzed the protein expression of a TGFβ signaling downstream effector, SMAD5, through ALK1 receptor activity, as well as TGFβR1 and SMAD2/3, the TGFβ alternative pathway. Only treatment with compound C changed the expression of phosphorylated (active) SMAD5 and total SMAD2/3 (Fig .5B).

## Discussion

Vascular complications are a major feature in diabetes. Metformin is largely used in diabetes treatment for its ability to control metabolism through AMPK. However, it is not well established whether modulating AMPK affects the angiogenic process within endothelial cells. Herein, we examined the effect of metformin on microvascular endothelial cell proliferation, invasion, migration and capillary-like structures formation, and compared this effect with two other AMPK modulators namely, AICAR and compound C. We were able to show that incubating HMECs with metformin under hyperglycemic conditions, leads to a significant reduction in the formation of capillary-like structures, as well as a significant reduction in migration and collagen production. Similar results were reported by Dallaglio et al. (12). By incubating HUVECs with different concentrations of metformin, these authors showed a significant dose- and time-dependent decline in the amount and length of segments of capillary-like structures. Metformin inhibitory effect was also reported in hepatic stellate cells (HSCs) activation, proliferation, migration and cell contraction (24).

Nevertheless, controversial findings have been described in the literature regarding the effect of metformin on cellular proliferation, apoptosis (21, 25) and angiogenesis under hyperglycemic conditions. Accordingly, metformin exhibited proangiogenic activity in experimental disease models like wound healing, cardiovascular disease and tumors (26, 27). In agreement, recent findings indicated that metformin improved angiogenesis and accelerated wound healing in diabetic mice, by promoting AMPK and eNOS signaling activity, often downregulated in diabetes. Cittadini et al. (26) investigated the effects of metformin in an experimental model of chronic heart failure, a common feature of diabetes, and observed a marked activation of AMPK with improved left ventricular remodeling, reduced perivascular fibrosis and minor cardiac lipid accumulation. Moreover, Bakhashab et al. (28) found that metformin can promote migration, inhibit apoptosis and increase the expression of VEGFA in HUVECs exposed to hyperglycemia-hypoxia condition.

Endothelial cells express two TβRI: ALK1 and ALK5, which present different affinities to both TGFβs and BMPs ligands. ALK1 binds with greater affinity to BMPs, whereas TGFβ preferentially binds to ALK5, enhancing angiogenesis. Since TGFβ expression was increased in diabetic kidney endothelial cells in previous studies of our group (29), and given the fact that metformin resulted in reduced angiogenesis, we next examined TGFβ/ALK5 signaling. Strikingly, ALK5 and SMAD2/3 expression was not affected by metformin. According to Iwata et al, metformin increased expression of SMAD6, an inhibitor of SMAD5 phosphorylation in human granulosa KGN cells (30). This inhibitory effect was further corroborated by the fact that Kdr did not significantly change after metformin treatment. 

TGFβ plays an important role in collagen synthesis, another process exacerbated during diabetes. TGFβ regulates the transcription of genes responsible for extracellular matrix components synthesis (31). Accordingly, in our experiment, a significant decrease in collagen synthesis was observed in metformin-treated HMEC under hyperglycemic conditions, which is in agreement with a putative inhibition of TGFβ signaling through SMAD6 upregulation by metformin.

To further analyze whether metformin effects were AMPK-dependent, we examined the action of AICAR, an AMPK agonist, and the AMPK inhibitor, compound C. AICAR is an adenosine analogue compound that has been extensively used to activate AMPK pathway *in vitro*. Its effects and efficacy vary according to the established cell culture conditions (32).

By stimulating AMPK activity, AICAR has been reported to prevent cell proliferation, migration, invasion and metastasis in several types of tumors both *in vivo* (33, 34) and *in vitro* (35-37). On the other hand, compound C, a pyrazolopyrimidine derivative, is a widely used potent inhibitor of AMPK. Nonetheless, it was shown to exert anti-proliferative effects and inhibit ICAM-1 and VCAM1 expression in cell and animal models to a similar extent as metformin (38).

Despite AICAR and metformin are both AMPK agonists, in the current study, they exhibited distinct effects in terms of angiogenesis. AICAR did not change migration, invasion or vessel assembly in HMECs. In addition, compound C, the AMPK antagonist, resulted in effects similar to those of metformin. These findings suggest that the anti-angiogenic action of metformin is probably not mediated via AMPK signaling pathway. In fact, metformin is known to interfere with several other metabolic pathways and present AMPK independent effects. A recent study showed that metformin suppresses adipogenesis in C3H10T1/2 MSCs by inhibiting mTOR/ p70S6k signaling pathway (39). According to Rena et al, metformin inhibits fructose-1,6-bisphosphatase, an enzyme implicated in glucose metabolism, through an AMPK-independent mechanism (40). These findings emphasize the hypothesis that the anti-angiogenic activity of metformin is AMPK independent. 

Altogether, our findings suggest that the antiangiogenic effects of metformin are AMPK-independent. Nevertheless, further studies are needed to confirm whether these activities involve the complex TGFβ signaling pathways.

## Conclusion

The present study shows that metformin not only regulates metabolism, but also probably controls endothelial dysfunction, being important in preventing conditions where angiogenesis is exacerbated such as diabetic retinopathy or nephropathy. 
